# Isolation, Culture and Comprehensive Characterization of Biological Properties of Human Urine-Derived Stem Cells

**DOI:** 10.3390/ijms222212503

**Published:** 2021-11-19

**Authors:** Martina Culenova, Andreas Nicodemou, Zuzana Varchulova Novakova, Michaela Debreova, Veronika Smolinská, Sona Bernatova, Dana Ivanisova, Olga Novotna, Jaromir Vasicek, Ivan Varga, Stanislav Ziaran, Lubos Danisovic

**Affiliations:** 1 Institute of Medical Biology, Genetics and Clinical Genetics, Faculty of Medicine, Comenius University, Sasinkova 4, 811 08 Bratislava, Slovakia; martina.culenova@fmed.uniba.sk (M.C.); andreas.nicodemou@fmed.uniba.sk (A.N.); zuzana.varchulova@fmed.uniba.sk (Z.V.N.); veronika.smolinska@fmed.uniba.sk (V.S.); sona.bernatova@fmed.uniba.sk (S.B.); danaivanisova@gmail.com (D.I.); 2 Panara Ltd., Krskanska 21, 949 05 Nitra, Slovakia; 3 National Institute of Rheumatic Diseases, Nabrezie I. Krasku 4, 921 12 Piestany, Slovakia; mdebre-ova@gmail.com (M.D.); ivan.varga@fmed.uniba.sk (I.V.); stanoziaran@gmail.com (S.Z.); 4 Department of Pediatric Urology, Faculty of Medicine, Comenius University, Limbova 1, 831 01 Bratislava, Slovakia; gonscakova1@uniba.sk; 5 Institute of Farm Animal Genetics and Reproduction, NPPC-Research Institute for Animal Production in Nitra, Hlohovecka 2, 951 41 Luzianky, Slovakia; jaromir.vasicek@nppc.sk; 6 Institute of Biotechnology, Faculty of Biotechnology and Food Science, Slovak University of Agriculture in Nitra, Tr. A. Hlinku 2, 949 76 Nitra, Slovakia; 7 Institute of Histology and Embryology, Faculty of Medicine, Comenius University, Sasinkova 4, 811 08 Bratislava, Slovakia; 8 Department of Urology, Faculty of Medicine, Comenius University, Limbova 5, 833 05 Bratislava, Slovakia

**Keywords:** urine-derived stem cells, biological characteristics, morphology, differentiation capacity, stemness, cytokine profile

## Abstract

Mesenchymal stem cells (MSCs) represent an attractive source within the field of tissue engineering. However, their harvesting often requires invasive medical procedures. Urine-derived stem cells (UDSCs) display similar properties to MSCs, and their obtention and further processing is non-invasive for the donors as well as low cost. Here, we offer a comprehensive analysis of their biological properties. The goal of this study was to analyze their morphology, stemness, differentiation potential and cytokine profile. We have successfully isolated UDSCs from 25 urine samples. First colonies emerged up to 9 days after the initial seeding. Cell doubling time was 45 ± 0.24 SD, and when seeded at the density of 100 cells/cm^2^, they formed 42 ± 6.5 SD colonies within 10 days. Morphological analyzes revealed that two different types of the cell populations have been present. The first type had a rice-grain shape and the second one was characterized by a polyhedral shape. In several cell cultures, dome-shaped cells were observed as well. All examined UDSCs expressed typical MSC-like surface markers, CD73, CD90 and CD105. Moreover, conditioned media from UDSCs were harvested, and cytokine profile has been evaluated showing a significantly higher secretory rate of IL-8, IL-6 and chemokines MCP-1 and GM-CSF. We have also successfully induced human UDSCs into chondrogenic, osteogenic and myogenic cell lineages. Our findings indicate that UDSCs might have immense potential in the regeneration of the damaged tissues.

## 1. Introduction

The functional urinary tract plays crucial role in maintaining physiological internal environment of the human body. One of its major functions involves urine production together with its excretion [1]. Urine is a liquid product made by a complex filtering process in the kidneys. It consists mainly of water, salts, electrolytes and other chemical compounds such as urea and uric acid [2]. The composition of urine is vastly affected by components in the circulating blood. That is why proper urine voiding is crucial for delivery of any toxic metabolites from the human body. From the scientific point of view, urine is no longer considered just a waste material. Microscopic analysis of urine sediment reveals that along with the crystals and casts, cells can be also commonly found [3]. In 1972, Southerland and Bain were the first ones to describe cells presence in the urinary sediment. Since then, more studies carried out results about successful isolation of urinary epithelial cells, but in 2008, Zhang et al. [4] pointed out that among multiple cell populations presented in the voided human urine, those with the stem cell characteristics could be found as well.

In constantly stressed tissues and organs, mesenchymal stem cells (MSCs) ensure proper regeneration and maintenance of the physiological functions [5]. They are generally described as the cells with unique self-renewal abilities which can easily adhere to tissue culture dishes, express specific cell surface markers and can be differentiated into more than one specific cell lineage [6]. Tissue biopsy presents traditional way of their harvesting. Although this method is well-adapted, disadvantages as invasiveness and donor site morbidity led to focus on alternative stem cell sources, one of which is urine. Not only urine-derived stem cells (UDSCs) possess similar properties as MSCs, but they can be also easily harvested via spontaneous voiding as the flow of the glomeruli filtrate creates the shear stress which detaches cells from adjacent tissues of the genitourinary tract. This simple technique is reproducible, low-cost and, most importantly, does not harm donors [7]. It had been already described that up to 7000 cells, both viable or dead, could be collected in urine within 24 h [8]. UDSCs represented one of the cell subpopulations (2–7 cells/100 mL urine sample) originating from renal tubules or the kidney papilla [9]. Due to their origin, attention has been drawn to their application within the field of tissue engineering and regenerative medicine of the genitourinary system. On the other hand, their simple collection and further processing increased interest in their overall clinical application, e.g., treatment of the autoimmune diseases.

Immunomodulatory activity of MSCs is currently an attractive topic, which still needs to be clarified in more detail. More data would help to predict their therapeutic effect with higher relevance. At present, it is assumed that their main performing mechanism is associated with paracrine signaling. Secreted molecules trigger pathways regulating all important biological processes at the affected and adjacent sites, e.g., cell cycle, cell-cell interaction, extent of the inflammatory response, neo-vascularization, etc. According to our knowledge, the cytokine profile of the UDSCs is not extensively characterized. As potential candidates for stem cell therapy, their secretory activity needs to be clarified and better understood in order to ensure their proper application in clinical medicine [10,11].

The present study offers deeper insight into human UDSCs. We focused on their morphological characterization, typical biological behavior, stemness, secretory activity and differentiation capabilities.

## 2. Results

### 2.1. Isolation, Cell Proliferation Kinetics and Clonogenic Potential

**Figure 1:**
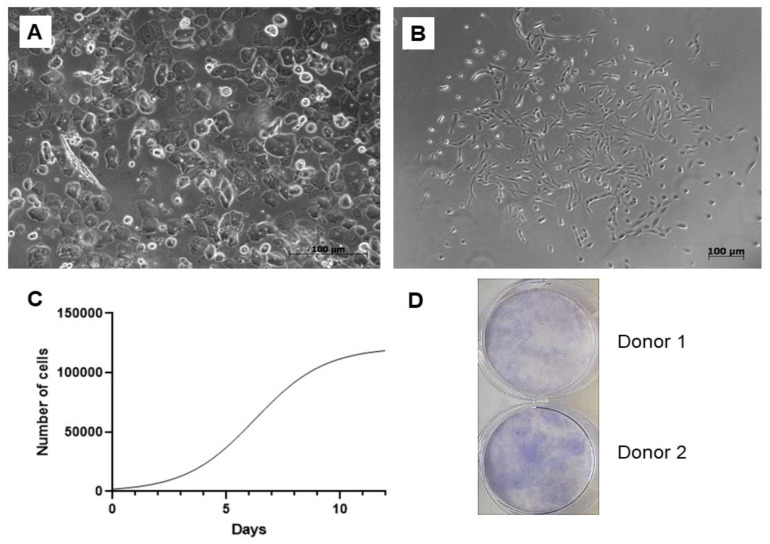
Isolation and basic biological characterization of the urine-isolated cells. (**A**) Image depicting freshly processed female urine sample. Multiple epithelial cells, forming non-adherent layer in a culture dish, could be detected. Non-adherent cells were removed after the first change of the culture medium. (**B**) Primary cell colonies observed after 5–9 days after initial seeding. (**C**) Representative growth curve of the isolated stem cells. Growth kinetics was studied over the period of 12 days. Exponential growth phase, in which cells proliferated rapidly, lasted for 6 days. (**D**) Representative figure of colony forming unit experiment. After 10 days, 42 ± 6.5 SD adherent cell colonies were observed.

A total of 25 urine samples were obtained from 10 adult volunteers, 4 women and 6 men aged between 25–45 years with no history of chronic illness or urinary tract infection symptoms. The success rate of the cell isolation was approximately 80%. Numerous epithelial cells could be microscopically observed immediately after the cell isolation. On the cellular level, sex differences have been clearly visible since epithelial cells were present in a higher rate within the samples of the females (Figure 1A). However, these non-adherent elements were removed as the medium was replaced for the first time. First, cell colonies emerged within 5–9 days, and larger colonies were formed up to 10–14 days (Figure 1B). The study of cell proliferation kinetics allowed us to assemble the graph of cells’ growth curve (Figure 1C). Its analysis revealed that the initial lag phase lasted for 3 days. Thereafter, cells entered an exponential growth phase which was present for 6 days. Stationary phase was attained at day 9. Cell doubling time was 45 ± 0.24 h. To evaluate clonogenicity of the UDSCs, a colony forming unit (CFU) assay was conducted. After 10 days of the culture, primary seeded single cells were able to form multiple adherent cell colonies (Figure 1D).

### 2.2. Analysis of the Cell Morphology

**Figure 2:**
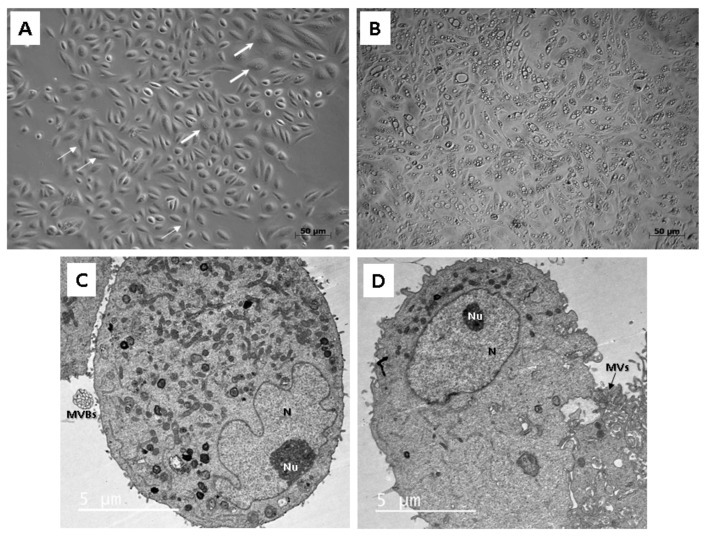
Morphological analysis of the human UDSCs. (**A**) Two different cell morphologies could be microscopically observed within one sample: rice-grain shape (thin arrows) and polyhedral-shape (thick arrows). (**B**) Image of the cell culture depicting dome-like structures, which could be spontaneously formed in confluent cell populations. These vesicles are filled with liquid and are capable of enlarging their volume, which could lead to the bursting of cells. (**C**) electron micrograph showing proteosynthetically and metabolically active cell with presence of endoplasmic reticulum, mitochondria and lysosomes. Multivesicular bodies (MVBs) were clearly visible as well. (**D**) Electronogram of the second type of UDSCs. Cytoplasmic organelles were mostly absent, which might have indicated the inactive form of the cell population.

To study the morphology, light as well as transmission electron microscopy (TEM) were applied. Throughout our experiment, we observed that isolated cells within 1 sample often did not display a uniform structure. In general, cells formed 2 types of populations, so different morphologies could be distinguished, a rice-grain shape and a polyhedral shape (Figure 2A). Colonies consisting of the rice grain-shaped cells proliferated rapidly. On the contrary, cells showing polyhedral morphology lagged in the growth and could not form such big populations as the other ones. Microscopic analysis also revealed the presence of the dome-like structures (hemicysts), which could be observed within several cell colonies (Figure 2B). However, this finding also indicated an ongoing process of senescence characterized by decreased proliferation rate and significant morphological alterations in particular cell populations.

Two different cell types could be also clearly distinguished ultra-structurally by applying TEM. Both types were mainly round or oval in shape. The cells adhered to the bottom of a Petri dish with numerous protrusions of cell membrane: microvilli and filopodia. The ultra-structural morphology of the first type reflected proteosynthetic and metabolic activity (Figure 2C). Each cell contained a large and pale nucleus, often eccentrically located with irregularly shaped deep indentations (increase of the nuclear surface) and a large amount of euchromatin. The nucleus had noticeable nucleolus located nearby the nuclear envelope. Abundant cisterns of rough endoplasmic reticulum, numerous mitochondria and membrane-bounded vesicles (or scattered, lysosome-like organelles) as well as granules of glycogen and lipid droplets were present in their cytoplasm. All these ultra-structural features are typical for MSCs. Moreover, the first type of cells created and secreted multivesicular bodies in cell membrane bounded amorphous electron-lucid vesicles. These secretory vesicles had around 0.5 µm in diameter. We assume that it could be a special type of communication between cells, probably the paracrine type of cell signaling. The second type of cells had a spherical, less euchromatic nucleus with a nucleolus, but there were almost no cellular organelles in the cytoplasm (Figure 2D). However, we do not rule out that both cell types are the same, only at different stages of development and activity. While the first type is protheosynthetically and metabolically active, the second may be an inactive, dormant form of the same cell population.

### 2.3. Phenotypic Analysis

**Figure 3:**
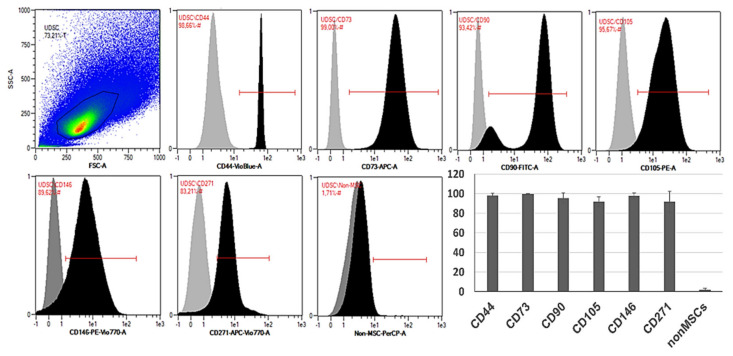
Representative histograms of the specific surface markers by flow cytometry. Isolated human UDSCs expressed typical MSC-like surface markers. Statistically, the highest expression was detected within CD73 (99.65% ± 0.38 SD), followed by CD44 (98.05% ± 2.37 SD), CD146 (97.91% ± 3.01 SD), CD90 (95.42% ± 5.44 SD), CD105 (91.77% ± 4.93 SD) and CD271 (91.72% ± 10.68 SD). The positivity for non-mesenchymal and hematopoietic surface markers (CD14/CD20/CD34/CD45 cocktail) was approximately 1.76% ± 1.57 SD.

Flow cytometric analysis confirmed high expression of typical MSC-like markers CD73, CD90 and CD105. UDSCs were also positive for CD44, CD146 and CD271. All examined cell specimens displayed low expression of non-mesenchymal markers together with hematopoietic CD markers such as CD14, CD20, CD34 and CD45 (Figure 3).

### 2.4. Cytokine Secretion Analysis

**Figure 4:**
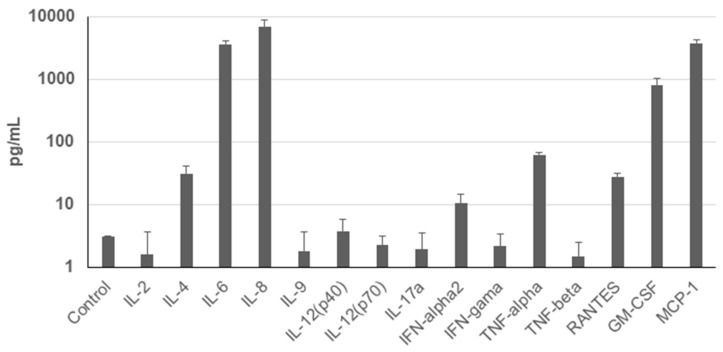
Cytokine profile of the human UDSCs. Analysis of the cells’ secretory activity revealed that IL-8 was secreted at the highest concentration (6958.33 pg/mL) followed by MCP-1 (3789.54 pg/mL), IL-6 (3650.03 pg/mL), and GM-CSF (817.89 pg/mL). Moderate levels within conditioned media were measured for TNF-alpha (62.4 pg/mL), IL-4 (31.29 pg/mL), RANTES (27.99 pg/mL) and INF-alpha2 (10.35 pg/mL). Concentration of other cytokines and chemokines was lower compared to the control (unconditioned) medium.

To assess the secretion profile of UDSCs, analysis of the conditioned media for selected cytokines and chemokines was performed. Results revealed significantly higher secretion rate of IL-8, MCP-1, IL-6, and GM-CSF. Moderate levels of TNF-alpha, IL-4, RANTES, and INF-alpha2 were detected as well. Concentration of other analyzed cytokines and chemokines e.g., IL-2 did not even reach the concentration of the control (unconditioned) medium (Figure 4).

### 2.5. Multilineage Differentiation

Isolated UDSCs were induced to differentiate into chondrogenic, osteogenic, adipo-genic and smooth muscle cell lineages under specific culture conditions.

**Figure 5:**
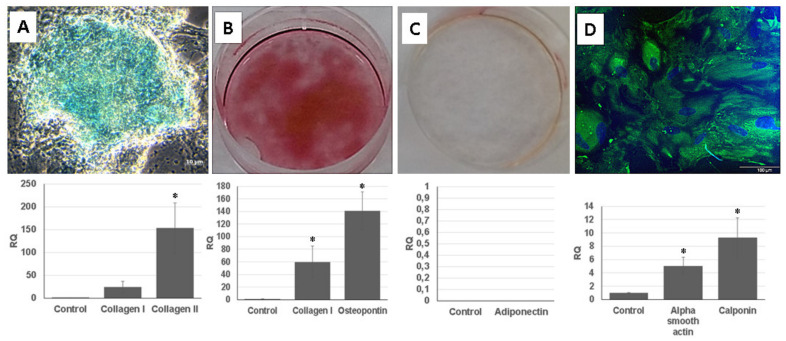
Multilineage differentiation of human UDSCs. (**A**) Chondrogenic differentiation. Image depicting glycosaminoglycan deposits which were formed after 28 days of induction in specific differentiation media. Results obtained from RT-PCR also confirmed high expression of collagen I and collagen II (* *p* < 0.05). (**B**) Alizarin Red was applied to stain calcium nodules and thereby determine the successful osteogenic induction. RT-PCR analysis revealed significantly higher expression levels of collagen I and osteopontin when compared to the control group (* *p* < 0.05). (**C**) Oil Red O staining together with RT-PCR were applied in order to assess successful adipogenic differentiation. However, isolated UDSCs did not differentiate into this cell lineage. (**D**) UDSCs induced into smooth cell lineage. Specific marker myosin (green) was confirmed by immunofluorescence. Nuclei were stained with DAPI. Moreover, significantly higher expression of alpha smooth actin and calponin I was detected as well (* *p* < 0.05).

For the chondrogenic differentiation, UDSCs were cultured in chondrogenic induction medium. After 28 days of induction, glycosaminoglycan deposits were found in the cell cultures and were also positively stained by Alcian Blue. 3D cell cultures were used for a real-time polymerase chain reaction (RT-PCR) analysis. Results proved chondrogenesis-related expression of genes encoding collagen I and collagen II (Figure 5A).

Calcium nodules, which were positively stained with Alizarin Red solution, could be found in the UDSCs cell cultures with osteogenic induction medium. Successful osteo-genic differentiation was also determined by RT-PCR test (Figure 5B).

UDSCs were treated by adipogenic differentiation medium for 24 days. After the experiment had been terminated, Oil Red O staining did not confirm the presence of intra-cytoplasmic lipid droplets in the UDSCs. RT-PCR analysis also did not show the expression of the adiponectin (Figure 5C).

For the smooth muscle induction, UDSCs were incubated in differentiation medium for 2 weeks. After 7 days of the culture, morphological changes could be observed, as induced cells became elongated and spindle shaped compared to the control group. RT-PCR test also confirmed successful differentiation as the expression of genes coded for actin alpha 2, and calponin I was detected. In addition, green signal for smooth muscle specific marker myosin was identified using immunofluorescent staining (Figure 5D).

## 3. Discussion

Kidney had been considered as an organ with non-regenerative capability [12]. However, great interest in the research of the UDSCs changed the point of view on this statement. Many studies had already described the UDSCs as a cost-effective and patient-friendly cell source applicable not only in the field of regenerative medicine of the urogenital system. According to our results, we can confirm the easy processing of the urine samples followed by the cell isolation. Regarding the optimization of the urine specimens’ volume, effective cell isolations had been observed if the volume was higher than 200 mL. In addition, when comparing the success rate of the cell harvesting between male and female donors, UDSCs showed higher cell yield in the male donors. However, this finding is not statistically confirmed. As described in previous studies, isolated cells adhered well on the plastic and reached 80% confluence up to 2 weeks. Nevertheless, there are also studies describing even higher proliferation rate as the same confluence was reached up to 3 days [13]. This shortened interval would be more beneficial for application in clinical medicine, as it could remarkably fasten the whole process of receiving an autologous cell therapy.

Clonogenicity is an important property of stem cells. Based on the results of the CFU assay, UDSCs are capable of forming a large population from a single cell. This indicates that just a one urine sample could be sufficient for maintaining a relevant amount of the stem cells without any rapid surgical intervention.

Microscopic analysis revealed that cells isolated from the fresh urine samples do not form homogenous cell colonies. These could be observed separately, or within one well in the plastic culture dish. According to our findings, at least two non-identical types of cell populations with different morphologies and proliferation kinetics could be distinguished. Their common property was the plastic adherence. The first type had regular, rice-grained shape; formed homogenous confluent colonies and proliferated rapidly. The second type displayed a flattened, polyhedral shape, and the proliferation rate was limited. In general, both types of cells maintained their morphology in the earlier stages of passages (up to Passage 5). However, structural changes could be visible within the higher numbers of the passages. Structural analysis applying TEM also confirmed the presence of two different cell types. The first type exhibited the typical ultrastructure of stem cells—nucleus highly rich in euchromatin with multiple indentations and numerous organelles in the cytoplasm. Similar morphological features could be also seen in other types of the stem cells, e.g., adipose tissue-derived stem cells, skeletal muscle-derived stem cells, and dental pulp-derived stem cells, as well as in human neonatal fibroblasts after in vitro cultivation [14,15,16]. Interestingly, we have also observed the formation of extracellular vesicles within this type of UDSCs. We assume they play role in paracrine secretion. The second cell type of UDSCs contained only few cytoplasmic organelles together with euchromatic nucleus, which had the spheroid shape and lacked the indentations. These ultrastructural properties could be also found in basal urothelial cells derived from the urinary bladder. Mentioned similarities were also reported by Polák et al. [17]. Several studies also identified two types of different cell types, which could be isolated from the urine sediment [18]. The first type was characterized as spindle-shaped, and the second type was referred to as the rice-shaped. Moreover, Dörrenhaus et al. [19] revealed in their study the formation of the domes, or hemicysts in cell populations derived from the urine samples. Independently from the morphology, dome-like structures were present in some of our cell cultures as well. These structures represent multicellular vesicles filled with the fluid which could gradually increase their volume. The final stage is characterized by the bursting. They are formed spontaneously in confluent cell populations. One study reported their presence in the cell culture of differentiated kidney epithelial cell lines as the proof of the transepithelial transport phenomena and claimed that this event is characteristic for the epithelial cell lines [20]. Related to our observations, the presence of the dome-like structures was followed by the senescence of that particular cell culture.

The first type of UDSCs, which displays characteristics typical for stem cells also at the ultrastructural level, can produce specialized extracellular vesicles. They represent special clusters of endomembrane vesicles enclosed by the plasma membrane (multivesicular bodies). They were firstly described in telocytes by Fertig et al. [21] and were named multivesicular cargos. Additionally, we have also detected similar multivesicular cargos, which were observed in stem cells derived from the dental pulp [22]. These structures play a fundamental role in paracrine communication, and apart from the stem cells, they can be also observed in endothelial cells derived from the diabetic kidney [23].

The examination of the surface markers is crucial for establishing the origin of the cells and also represents the gold-standard in stem-cell research. However, outcomes from multiple studies focusing on the phenotypization of the UDSCs often produced ambiguous data [18]. According to our findings, frequently, the highest expression was observed for CD44, CD73 and CD146, where the cell positivity was more than 95%. In addition to these data, CD73 appeared to be the most stable marker, as its expression in all examined cultures was higher than 99% (99.65 ± 0.38 SD), followed by CD44 (98.05% ± 2.37 SD) and CD146 (97.91% ± 3.01 SD). Similar results were also described in recent studies [5,13,24]. Moreover, strong expression of CD44 and CD146 was linked with the urothelial basal cells and pericytes, which underlined their urogenital origin [25]. CD271 also complements the broad scale of CD markers typical for the multipotent MSCs derived from various tissues [26]. This marker was present in our cultures with average frequency of 91.72% ± 10.68 SD. The presence of other characteristic MSC-like markers (CD90, CD105) differed within samples. When compared with other studies, part of them described their frequency as high (> 97%) [25,27]. However, publications in which lower expression rate was pointed out could be found as well [5,24]. Performed flow cytometric analyzes of our cell cultures revealed that the percentage of positivity for CD90 and CD105 was in average 95.42% ± 5.44 SD and 91.77 ± 4.93 SD, respectively. Precise investigation of the phenotype can also elucidate specific pathways regulating the typical properties of the stem cells such as self-renewal or multipotent differentiation capability. UDSCs fulfilled the phenotypic requirements for identifying them as the stem cells. However, our outcomes indicated the non-uniform expression of the specific CD markers as well as multiple cell types isolated from human urine. This might greatly affect not only the biological behavior of UDSCs but also their further application as a cell therapy.

The therapeutic effect of the MSCs is traditionally assigned to their paracrine activity [28]. A broad spectrum of immunomodulatory molecules with immune-suppressive effects has been described, e.g., TGF-ß, IL-10, hepatocyte growth factor (HGF), indoleamine 2,3-dioxygenase (IDO), prostaglandin E2 (PGE2), etc. Paradoxically, UDSCs secretome has not been sufficiently characterized. Several studies described immunomodulatory effect of exosomes derived of UDSCs [29,30,31]. Here, we performed the analysis of the secretion of the basic cytokines and chemokines under standard in vitro conditions. Our data revealed significantly increased secretion of 3 particular molecules—IL-6, IL-8 and MCP-1. In general, IL-6 plays essential role in anti-inflammatory effects of MSC by stimulating local lymphocytes [11,29]. Moreover, IL-6 stimulated MSCs can in turn activate Treg cells, which are known for their immunosuppressive capacity and immune system modulation. Similar mechanism is also expected to operate in UDSCs. Higher secretion of IL-8 could be important in the context of the UDSCs usage in the tissue engineering. This chemokine seems to promote the angiogenesis through increased secretion of vascular endothelial growth factor. The experiment was performed on the bone marrow stem cells [32]. In the field of the tissue engineering, scaffolds seeded with differentiated stem cells display immense potential to repair various tissues. For these cell-seeded constructs, it is crucial to obtain sufficient neo-vascularization when applied in vivo. UDSCs seem to be a good candidate for this type of application due to their high IL-8 secretion. In addition, Li et al. [33] associated the lower secretion of IL-8 in cell cultures of placenta-derived MSCs with an early cell aging. In our experiments, we have observed that cultures in which multiple hemicysts were found, the premature senescence occurred. To better understand this phenomenon, measurement of the IL-8 levels in particular cell populations with hemicysts could be beneficial. MCP-1 is considered as another significant chemokine secreted by MSCs [34]. UDSCs secreted significantly higher levels of this chemokine. MCP-1 affects several cell types which are critical for the immune system, mainly monocytes and macrophages. Moreover, this chemokine potentiates the transition of macrophages from pro-inflammatory into anti-inflammatory phenotype which underlines the regenerative potential of the stem cells when applied into the affected site both under in vitro and in vivo conditions [35,36]. Similarly, a recent study carried out by Cao et al. also revealed that alveolar bone-derived stem cells secreted mainly IL-6 and MCP-1, and thus, at least partially, explained mechanisms behind their immunosuppressive capacity [37]. Intriguingly, IL-6 was also de-scribed as a part of extracellular vesicles derived from the UDSCs [29]. Moreover, these vesicles carried multiple bioactive molecules which could stimulate B-Lymphocytes and simultaneously suppress the function of T-Lymphocytes. This evidence could be promising especially in the clinical medicine in the treatment of immunodeficiency or oncogenic diseases. All mentioned findings underline the great potential of the UDSCs to be applied as a cell therapy for various diagnosis, but primary for those where suppression of the immune system and attenuation of pro-inflammatory pathways are the main triggers of the pathological mechanisms. Therefore, it is important to understand the immunomodulatory potential of UDSCs in standard in vitro conditions as it can help to predict their behavior in vivo. However, for future experiments, it will be necessary to analyze more immunomodulatory molecules secreted by UDSCs to obtain complex information about their secretory capacities. Importantly, these types of experiments need to be performed not only under basal in vitro but also pathological conditions to capture the influence of abnormal microenvironment on cell secretion.

Multipotent differentiation capacity of the UDSCs has been already broadly investigated [18,38,39]. Various studies described the successful transition into mesenchymal derivates, cell lineages of the urinary, neural, endothelial or myogenic system [18]. In the present manuscript, we have focused on the UDSCs induction into osteogenic, chondro-genic, adipogenic and myogenic cell lineages. The positivity of the experiments was determined by standard histological/immunofluorescent staining together with RT-PCR. Out of the mentioned inductions, only 3 were successful (osteogenic, chondrogenic and myogenic). UDSCs repeatedly failed in obtaining the adipogenic phenotype despite of the fact that we optimized the composition and concentration of supplements and tested at least 4 different types of media. We have also tried to differentiate UDSCs into uroepithelial cells. However, the induced cells did not acquire the expected traits either. We tried to optimize several steps. First of all, only cell cultures with suitable phenotypic profile were selected. As mentioned above, heterogenous cell populations with different proliferation kinetics can be harvested from the urine sample, and therefore, the isolated polyhedral cells, which seemed to have inferior biological properties when compared to rice-grain shaped cells, could alter the frequency of expression of the surface CD markers and possibly inhibit the differentiation process. The next step included the optimization of the cell density together with the composition of the differentiation media, both commercial and self-mixed were used. Finally, we tried to adjust the duration of the incubation. The mentioned processes helped us to obtain better results in osteogenic, chondrogenic and myogenic differentiation. Unfortunately, no changes were observed within adipogenic or uroepithelial induction. Few publications can be found to describe an unsuccessful or deficient differentiation experiments as well [5,40]. For the future experiments, we want to apply the co-cultivation technique or use conditioned media to obtain positive results.

Although multiple studies pointed out the potential of the UDSCs to be applied in the stem-cell therapy of various diseases, most of the studies were performed in laboratories or on the animal models. The translation into clinical medicine is still in its infancy, highlighting the high demand for the further research. It would be also beneficial to identify and separate the exact population isolated from the urine sample which mostly resembles the characteristic properties of MSCs. On the other hand, non-invasiveness and cost-effectiveness of the UDSCs harvesting still belong to the biggest benefits as the donors’ safety and comfort are essential for successful cell therapy.

## 4. Materials and Methods

### 4.1. Isolation and Cultivation

Urine samples were obtained from 10 healthy adult volunteers (6 men, 4 women) via spontaneous voiding. The age range of the donors was 25–45 years, and they had no history of any severe systematic disease. Mid- and last-stream urine was collected into sterile 50 mL centrifuge tubes, whereas the overall volume of the samples was up to 200 mL. Most samples were processed immediately or within 4 h after the collection. Isolation consisted of the following 3 steps: centrifugation, washing and seeding. Briefly, samples were centrifuged at 500× *g* for 10 min at room temperature. Afterwards, the supernatants were carefully discarded, leaving approximately 1 mL of the pellet in the centrifugation tubes. Next, 5 mL of phosphate buffer saline (PBS, Sigma-Aldrich, St. Louis, MO, USA) was added to wash the cell pellets, followed by the second centrifugation (500 g for 10 min), and supernatants were gently removed leaving approximately 800 μL of the pellets in the tubes which were subsequently resuspended in the primary culture medium. Cell suspensions were then plated in a 24-well plate and incubated at 37 °C in a humidified atmosphere with 5% CO_2_. The primary culture medium consisted of embryonic fibroblast medium (EFM) and keratinocyte serum free medium (KSFM, Cell Applications, San Diego, CA, USA) at the ratio 1:1, supplemented with 5% fetal bovine serum (FBS, PAN-Biotech, Aidenbach, Germany) and antibiotics Pen/Strept (Sigma-Aldrich, St. Louis, MO, USA). We also applied other supplements such as Renal Epithelial Growth Medium SingleQuots^TM^ Kit (REGMTM, Lonza, Basel, Swiss) [6] and ROCK-Inhibitor (Sigma-Aldrich, St. Louis, MO, USA) with a final concentration of 10 μM. The primary medium was changed after 72 h with the fresh proliferation medium, and then, the changing of the medium was performed every 3 days. After reaching 80% confluency, cells were passaged using 0.05 % trypsin (Sigma-Aldrich, St. Louis, MO, USA). Table 1 and Table 2 summarize all components of both media that were used for the cell culture. 

### 4.2. Cell Proliferation and Colony Forming Units

To assess the proliferation kinetics, UDSCs (Passage 4) were seeded in 24-well plates at a density of 4000 cells/well. Afterward, cells were counted at the pre-determined time intervals (days 1, 3, 5, 7, 9 and 12) by using CEDEX XS Cell Analyzer (Roche, Switzerland). In brief, at the estimated day, cells were trypsinized, put into sterile 15 mL centrifugation tubes and subsequently centrifuged for 9 min at 1200 rpm at the room temperature. Redundant supernatant was carefully aspirated leaving only 1 mL of cell suspension in the tubes. Next, 50 µL of the suspension was immediately stained by trypan blue and put into an 8-channel cell counting chamber. Cell doubling time was counted using the following equation: Cell Doubling Time = duration of cell culture × log2/log (final concentration)−log (initial concentration). To obtain statistically relevant data, all measurements were performed three times.

The CFU assay was performed in order to estimate the clonogenicity of the UDSCs. Isolated cells from passages 3–4 were plated on a 6-well plate at a density of 100 cells/cm^2^ in a proliferation medium and incubated under standard conditions for 10 days. Afterward, cell cultures were washed by PBS, fixed with 4% formaldehyde and stained with 0.5% crystal violet. Colonies bigger than 2 mm were manually counted. The experiment was performed in triplicate.

### 4.3. Morphological Analysis

The structure of the UDSCs was analyzed under light as well as TEM.

To observe cell cultures under the light microscope, a Zeiss Axiovert 100 (Carl Zeiss, Jena, Germany) was used, and cell cultures of various passages (Passage 1–5) were studied using various magnifications (40×, 100×, 200×).

The sample preparation for the TEM consisted of several steps. At first, 10,000 cells (Passages 3–5) were collected by trypsinization and subsequently fixed by freshly prepared 3% glutaraldehyde in 0.2 M PBS at room temperature for 30 min. Then, a cell pellet was post-fixed using 1% osmium tetroxide solution for 1 h. Followed by dehydration in ascending ethanol concentrations (10–100%), the samples were embedded into resin and subsequently cut using ultramicrotome. Ultra-thin sections were then mounted on the copper grid and observed. Analysis was performed on JEOL JEM-2100 (JEOL, Tokyo, Japan).

### 4.4. Flow Cytometry Analysis

Immunophenotypic analyses with flow cytometry were performed according to manufacturer’s recommendations. Briefly, 1 × 10^6^ cells per sample (Passage 3–4) were centrifuged at 300× *g* and resuspended in 100 µl of buffer. Afterwards, 10 µl of the respective antibodies, CD44-VioBlue, CD73-APC, CD90-PE, CD105-FITC, CD146-PE-Vio770 and CD271-APC-Vio770 (Miltenyi Biotec, Bergisch Gladbach, Germany) and a cocktail of CD14/CD20/CD34/CD45-PerCP (Miltenyi Biotec, Bergisch Gladbach, Germany), was added to cell suspension and incubated for 10 min in the dark in the refrigerator. Then, cells were washed with 2 mL of buffer and centrifuged. Supernatant was aspirated, and the final sediment was resuspended in buffer for flow cytometry analysis. Similarly, respective iso-types controls were used to assess background fluorescence and non-specific binding of anti-bodies to cells. All data were acquired using a MACSQuant Analyzer 10 (Miltenyi Biotec, Bergisch Gladbach, Germany) and further analyzed by MACS Quantify software (Miltenyi Biotec, Bergisch Gladbach, Germany).

### 4.5. Assessment of the Cytokine and Chemokine Profile

The secretory function of UDSCs was estimated by applying MILLIPLEX MAP Human Cytokine/Chemokine Magnetic Bead Panel–Immunology Multiplex Assay (Merck, Darmstadt, Germany), and the measurement was performed on a Luminex MAGPIX^®^ Instrument (Luminex, Austin, TX, USA). For the determination of cytokine concentrations, triplicates of cell-free supernatants were collected 48 h after UDSCs were seeded at the density of 100 × 10^3^ cells in 6-well plates. Cells were kept in a serum-free medium consisting of equal volume of KSFM and DMEM high-glucose. Further, sample processing and measurement were performed according to manufacturer’s instructions. Data were analyzed and processed using Belysa^TM^ software (Merck, Darmstadt, Germany).

### 4.6. Differentiation Experiments

UDSCs at passage 4 were induced to differentiate into adipogenic, osteogenic, chondrogenic, urothelial and smooth muscle cell lineages.

For the adipogenic differentiation, 50 × 10^3^ cells per well were seeded into a 12-well culture plate in a pure DMEM high-glucose medium which did not contain any supplements. After 48 h, the medium was switched, and adipogenic differentiation medium was added. The differentiation medium consisted of the following reagents: DMEM high-glucose medium (Sigma-Aldrich, St. Louis, MO, USA) supplemented with penicillin/streptomycin, 10% FBS, 1 µM Dexamethasone (Sigma-Aldrich, St. Louis, MO, USA), 500 µM Isobutyl-1-methylxantine (Sigma-Aldrich, St. Louis, MO, USA) and 66 µM Indometacin (Sigma-Aldrich, St. Louis, MO, USA), 500 µM Hydrocortisone (Sigma-Aldrich, St. Louis, MO, USA). The differentiation medium was changed every 72 h, and cells were cultured for 24 days. After the termination of the experiment, samples were stained with Oil-Red O solution (Merck, Darmstadt, Germany) as well as processed for RT-PCR analysis. UDSCs cultured in medium consisting of DMEM-high-glucose medium and penicillin/streptomycin supplemented with 10% FBS were used as a control group.

For the chondrogenic induction, 2D and 3D cell cultures were established. For the differentiation of a 2D cell cultures, 50 × 10^3^ cells were plated in a 12-well culture plate and influenced by specific differentiation medium consisting of DMEM/F12 (Sigma-Aldrich, St. Louis, MO, USA) medium supplemented with 10% FBS, penicillin/streptomycin, 0.83 mg/mL NaHCO_3_ (Sigma-Aldrich, St. Louis, MO, USA), 6 µg/mL Insulin (Sigma-Aldrich, St. Louis, MO, USA), 0.2 mM Ascorbate-2-phosphate (Sigma-Aldrich, St. Louis, MO, USA), 10 ng/mL TGF-ß1 (R&D Systems, Minneapolis, MN, USA). The medium was changed every 3 days. The culture lasted for 28 days, and the results were evaluated via standard staining with Alcian blue (Merck, Darmstadt, Germany). To establish 3D cell cultures, 2.5 × 10^5^ cells in sterile conical 15 mL tube were centrifuged at 2000 rpm for 5 min and left overnight to form an aggregate in a proliferation culture medium. After 24 h, the medium was replaced with 0.5 mL of the chondrogenic differentiation medium, which was described above. The medium was changed every 72 h, and the experiment lasted for 28 days. RT-PCR was subsequently performed in order to assess the successful differentiation. Cells cultured in DMEM/F12 supplemented with 10% FBS, 0.83 mg/mL NaHCO_3_ and penicillin/streptomycin were used as a control group.

50 × 10^3^ of UDSCs were seeded in 12-well plates in order to assess their potential to differentiate into osteogenic cell line. After the initial seeding, the standard proliferation medium was replaced with a differentiation medium. It consisted of DMEM low-glucose (Sigma-Aldrich, St. Louis, MO, USA), 10% FBS, L-Glutamine, penicillin/streptomycin, 55 nM Dexamethasone, 775 µM Ascor-bate-2-phosphate and 10 mM Glycerophosphate (Sigma-Aldrich, St. Louis, MO, USA). The medium was changed every 72 h. The cells were cultured for 28 days, and after this time period, results were evaluated by staining using Alizarin red solution (Sigma-Aldrich, St. Louis, MO, USA) and RT-PCR analysis. UDSCs in the control group were affected by the following medium: DMEM-low glucose supplemented with 10% FBS, l-Glutamine and penicillin/streptomycin.

For the smooth muscle differentiation, cell density was 50 × 10^3^ cells per well (12-well culture plate). Differentiation medium contained DMEM high-glucose and EFM at the 1:1 ratio, 10% FBS, 2.5 ng/mL TGF-ß1 and 5 ng/mL PDGF-BB (R&D Systems, Minneapolis, MN, USA). The experiment lasted for 14 days. Cells used in differentiation experiments were at the passages 2–3. The differentiation medium was changed every 3 days. Cells seeded in DMEM high-glucose and EFM medium (1:1) supplemented with 10% FBS served as a control group.

### 4.7. RNA Isolation and Quantitative Real-Time PCR

RNA from the samples was isolated using the GeneJET RNA Purification Kit (Thermo Fischer Scientific, Waltham, MA, USA) according to manufacturer’s guidelines. Obtained RNA was further used to synthetize complementary DNA with Maxima First Strand cDNA Synthesis Kit (Thermo Fischer Scientific, Waltham, MA, USA). Subsequently, RT-PCR was performed using Eco Real-Time PCR System (Illumina, San Diego, CA, USA). Reactions were carried out as follow: 50 °C for 2 min, 40 cycles at 95 °C for 10 min, 95 °C for 15 s and finally 60 °C for 1 min. Quantification of following proteins was estimated: adiponectin, osteopontin, collagen I, collagen II, actin alpha 2 and calponin I. Human glyceraldehyde-3-phosphate dehydrogenase was used as an internal control. Relative expression levels of the selected genes were measured by the ∆∆ CT method.

### 4.8. Immunofluorescence

The successful specific differentiation into a smooth muscle cell line was also evaluated by assessing the presence of myosin in the cytoplasm of differentiated UDSCs. In brief, cells were grown on plastic coverslips and fixed with ice cold methanol for 5 min at −20 °C. Non-specific binding was blocked using 1% Bovine Serum Albumin (BSA, Sigma-Aldrich, St. Louis, MO, USA) in PBS for 30 min at 37 °C. Then, cells were incubated with primary antibody against myosin (Sigma-Aldrich, St. Louis, MO, USA) diluted 1:10 in PBS with 0.5% BSA (PBS/BSA) for 1 h at 37°C, washed four times with PBS containing 0.02% Tween 20 for 10 min followed by the incubation with fluorescent secondary antibody (donkey anti-rabbit Alexa Fluor 488; Thermo Fisher Scientific, Waltham, MA, USA) diluted 1:1000 in PBS/BSA for 1 h at 37 °C. Subsequently samples were washed once with PBS, incubated with DAPI (1:1000) in PBS to stain nuclei and washed three times with PBS for 10 min. The fluoroshield mounting medium (Sigma-Aldrich, St. Louis, MO, USA) was used to mount coverslips onto slides. Finally, the cells were analyzed by Zeiss Axioscope 5 fluorescent microscope.

### 4.9. Statistical Analysis

Quantitative data were presented as mean ± standard deviation (SD). One-way ANOVA followed by Bonferroni and Holm post hoc tests for multiple comparisons were used when appropriate, and *p* < 0.01 with *p* < 0.05 were considered as statistically significant. All experiments were performed in triplicate.

## 5. Conclusions

In conclusion, we have successfully isolated human UDSCs which share biological characteristics typical for multipotent mesenchymal stem cells. Moreover, their immunomodulatory activity supports their potential to be applied as a stem cell therapy. On the other hand, more research needs to be carried out under in vivo conditions in order to elucidate their real behavior in a pathological environment.

## Figures and Tables

**Table 1:** Composition and formula of the primary proliferation medium.

Reagent	Volume
KSFM medium (Cell Applications, San Diego, CA, USA)	250 mL
EFM medium single components:	
DMEM high-glc (Sigma-Aldrich, St. Louis, MO, USA)	217.5 mL
MEM Non-essential Amino Acid Solution (Sigma-Aldrich, St. Louis, MO, USA)	2.5 mL
Penicillin/Streptomycin 100× (Sigma-Aldrich, St. Louis, MO, USA)	2.5 mL
l-Glutamine (Sigma-Aldrich, St. Louis, MO, USA)	2.5 mL
FBS (PAN-Biotech, Aidenbach, Germany)	25 mL
REGM SingleQuots Kit (Lonza, Basel, Swiss)	3.5 mL
ROCK Inhibitor (Sigma-Aldrich, St. Louis, MO, USA)	10 μM

**Table 2:** Composition and formula of the proliferation medium.

Reagent	Volume
KSFM medium (Cell Applications, San Diego, CA, USA)	250 mL
EFM medium single components:	
DMEM high-glc (Sigma-Aldrich, St. Louis, MO, USA)	217.5 mL
MEM Non-essential Amino Acid Solution (Sigma-Aldrich, St. Louis, MO, USA)	2.5 mL
Penicillin/Streptomycin 100× (Sigma-Aldrich, St. Louis, MO, USA)	2.5 mL
l-Glutamine (Sigma-Aldrich, St. Louis, MO, USA)	2.5 mL
FBS (PAN-Biotech, Aidenbach, Germany)	25 mL

## Data Availability

The data used to support the findings of this study are available from the corresponding author upon reasonable request.
